# Effects of folic acid supplementation before conception on innate immunity and anti-HBs levels of offspring born to HBsAg-positive mothers

**DOI:** 10.3389/fnut.2025.1526053

**Published:** 2025-04-23

**Authors:** Jia Lian, Zhaoyue Men, Xiuyang Xu, Yandi Li, Jinbo Li, Weigang Wang, Tian Yao, Yuping Li, Yiqun Qu, Yongliang Feng, Suping Wang

**Affiliations:** ^1^Department of Epidemiology, School of Public Health, Shanxi Medical University, Taiyuan, China; ^2^Center of Clinical Epidemiology and Evidence Based Medicine, Shanxi Medical University, Taiyuan, China; ^3^Key Laboratory of Coal Environmental Pathogenicity and Prevention, Ministry of Education, Shanxi Medical University, Taiyuan, China; ^4^Reverse Etiology Research Center Academician Workstation, Shanxi Medical University, Taiyuan, China; ^5^First Hospital of Shanxi Medical University, Taiyuan, China; ^6^Department of Obstetrics and Gynaecology, The Third People's Hospital of Taiyuan, Taiyuan, China

**Keywords:** anti-HBs, cohort study, folic acid, HBsAg-positive mothers, innate immunity

## Abstract

**Introduction:**

Folic acid has been associated with fetal development, especially in fetal immunity. Therefore, limited evidence regarding the effects of different folic acid supplementation of hepatitis B surface antigen (HBsAg) positive mothers in innate immunity in offspring. Herein, this study aimed to explore the association between folic acid supplementation and the innate immunity of neonates and the immunological efficacy of hepatitis B vaccine (HepB), which may provide insights that could inform pre-pregnancy health management in HBsAg-positive mothers.

**Materials and methods:**

It is an ambispective cohort study with 293 pairs of HBsAg-positive mothers-offspring in Taiyuan, Shanxi Province, China. Mothers were classified into three groups according to the time of starting folic acid supplementation, non-supplementation group, pre-pregnancy group and post-pregnancy supplementation group. Immunological indexes such as immune cells proportion and innate immune mediators in cord blood and anti-HBs in infants were measured. Differences in immunological indexes were analyzed by One-Way ANOVA test. Univariate and multivariate analyses were performed for factors associated with abnormal immunological indexes and potential confounders were adjusted.

**Results:**

The preconception folic acid group showed a significantly higher expression levels of STING (*P* = 0.005) and pNF-κB (*P* = 0.010) in cord blood along with higher anti-HBs titres (*P* = 0.006), when compared to both non-supplementation group and post-pregnancy supplementation group. Higher anti-HBs levels indicate a stronger immune response to HepB and may enhance protection against HBV infection during early life. Infants in the high pNF-κB expression group exhibited a significantly elevated seropositive rate of HepB compared to those in the low pNF-κB expression group (*P* = 0.037). There were no mediation effects and no moderation effects in this study, potentially due to the direct influence of folic acid supplementation on immune responses or the limited sample size.

**Discussion:**

In conclusion, our findings demonstrate that preconception folic acid supplementation may enhance HepB vaccine responsiveness in infants of HBsAg-positive mothers. Meanwhile, high pNF-κB expression in cord blood can increase seropositive rates in infants. This discovery has significant public health implications, as it may provide a simple and accessible intervention to improve vaccination outcomes and reduce HBV transmission in endemic regions.

## 1 Introduction

Pregnancy, even before the onset of pregnancy, as a primary stage of life development for 10 months, has a crucial impact on infants development. Folic acid is an essential water-soluble vitamin that plays an essential role in cell growth and division and the maintenance of new cells. It is reported that folic acid contribute to immune function, immune response and immune-mediated diseases (1). In experimental animals, folic acid deficiency resulted in poor dendritic cell and spleen cell responses (cytokine production) and altered T cell phenotypes, and impairs antibody production (2), Folic acid supplements have been reported to increase some immune biomarkers, including those associated with anti-viral defense, and to decrease infections and immune-related proteins (3).

Hepatitis B virus (HBV) infection affects 300 million people globally (4), with mother-to-child transmission (MTCT) accounts for over one-third of chronic cases (5). High maternal HBsAg level may increase the risk of immunoprophylaxis failure in infants (6), underscoring the unique immune challenges in this population. While maternal folic acid supplementation can improve the persistence of protective antibodies (7), whether and how maternal folic acid supplementation affect HBsAg-positive mothers infants immune system remains unclear.

The umbilical cord serves as a critical connection between the fetus and mother. Umbilical cord blood, which is representative of neonatal blood, contains a rich source of immune cells and have many biological functions, such as anti-inflammation, and immune regulation (8). The proportion of immune cells in umbilical cord blood can reflect the immune status of infants to a certain extent.

Innate immunity is first line of host of defense, which can prevent infection and attack the invading pathogens (9). Innate immunity can distinguish structural components from microbial pathogens by a variety of proteins present in the host cells such as the pattern recognition receptors (PRRs). PRRs include TLRs, NOD-like receptors (NLRs), C-type lectin receptors (CLRs), and RIG-I-like receptors (RLRs) (10). Several molecules include the myeloid differentiation primary-response protein 88 (MyD88), TIR domain-containing adaptor-including interferon-β(TRIF) have been described participate in the process of TLRs binds to its ligand. Moreover, these adaptors mediate the activation of transcription factors such as the nuclear factor-κB (NF-κB) and the interferon regulatory factor (IRF) and induce the expression of inflammatory and anti-inflammatory cytokine and chemokine genes in turn (9, 11). Stimulator of interferon genes (STING) signaling pathway can produce protective immune responses against pathogens with a variety of DNA structures. When abnormal DNA including exogenous DNA produced by viruses and bacteria, such as HBV, is present in the cytoplasm, cGAS can recognize exogenous DNA and combine to synthesize cyclic GMP-AMP (cGAMP) and activate downstream STING. When it is activated, STING can recruit interferon regulatory factor 3 (IRF3) to phosphorylate to synthesize interferon. Moreover, NF-κB dimer can be activated by STING to produce inflammatory cytokines (12). Mitochondrial antiviral signaling protein (MAVS) functions as a platform for antiviral innate immune signal transduction (13). Upon viral infection, MAVS bind to cytosolic RNA sensor RIG-I and subsequently activates downstream NF-κB and IRF3/7-related signal pathways (14, 15). Innate immune mediators play a critical role in neonatal immunity. However, the influence of maternal folic acid supplementation on infants innate immune mediators has been neglected.

Hepatitis B (HepB) vaccination is the most effective way to prevent HBV infection and good responses to the HepB is a sign of good immune status. The production of hepatitis B surface antibody (anti-HBs) is the result of a combination of innate and adaptive immunity. HepB vaccine response is a continuation of neonatal immunization. It is reported that the recipients' response to the HepB may be influenced by their T-cell immunity (16). Moreover, low expression of neonatal innate immune mediators STING and pIRF3 and low percentage of plasma cells were risk factors of non/hypo-response to HepB in infants (17).

While folic acid supplementation is known to influence immune development, its specific effects in HBsAg-positive mothers and on infants innate immunity remains poorly understood. This study addresses a gap by investigating how preconception folic acid supplementation modulates both innate immunity and anti-HBs levels in offspring of HBsAg-positive mothers—a population at high risk of chronic HBV infection. Our findings may also provide actionable insights for optimizing prenatal guidelines in HBsAg-positive mothers.

## 2 Materials and methods

### 2.1 Study subjects

This ambidirection cohort study integrated prospective and retrospective data from November 2019 to January 2023. This study was approved by the Ethics Committee of Shanxi Medical University (No. 2018LL323, 9 March 2018). All participants provided signed informed consent before enrollment. From November 2019 to June 2022. A total of 293 HBsAg-positive mothers and their neonates were enrolled at the Department of Obstetrics and Gynecology of the Taiyuan Third People's Hospital, China. Baseline demographic and biological specimens were collected at delivery. Infants were followed up at 7–9 months age and their peripheral blood samples were collected to assess indicators of HBV infection status and evaluate the immune response to HepB.

The specific inclusion criteria are as follows: (1) The mother was HBsAg-positive; (2) The newborns was a single live birth without birth defects. The exclusion criteria are as follows: (1) mother infected with hepatitis C virus, HIV or other virus; (2) twins or multiple births; (3) neonates or infants were HBsAg-positive. The study was approved by the Ethics Committee of Shanxi Medical University. All participants provided written informed consent.

### 2.2 Demographic and obstetric data collection and sample collection

The demographic and obstetric data of mothers (including age, education level, gestational week and HBV infection status) and neonates (including sex, birth weight, birth length, and Apgar score) were collected by questionnaires survey and medical record review. The stage, frequency and dose of folic acid supplementation during 1 year prior to conception were also collected. Follow-up was conducted for infants for 1–3 months after the full vaccination of HepB. The follow-up data including HepB vaccination and feeding pattern were collected by in-person questionnaires and certificates of vaccination.

Before delivery, 5 ml of venous blood of each woman was collected and centrifuged at 1,000 rpm/min for 5 min, then the serum was obtained and stored in the refrigerator at −80°C for later use. Venous blood was collected from each neonate within 12 h before immunoprophylaxis and from every infant aged 7–9 months. 3 ml neonatal and infant blood was collected and placed in an anticoagulant tube. The plasma was obtained after centrifuge and stored in −80°C.

### 2.3 Anti-HBs measurement

Serum anti-HBs was detected by chemiluminescence microparticle immunoassay (CMIA). All samples were processed and determined strictly according to the manufacturer's instructions.

### 2.4 Flow cytometry

The key proteins of innate immune signaling pathway molecules in neonatal cord blood were detected by flow cytometry. For endoprotein staining, the cells need to be fixed by fixation buffer. After 20 min remove fixation buffer, cells were permeabilized by perm buffer. After 5 min remove perm buffer, the cell suspensions were incubated for 15 min at 4°C with Recombinant anti-MyD88 antibody (Abcam, ab133739) and after wash three times the cells were incubated for 15 min at 4°C with Goat Anti-Rabbit IgG H&L (Alexa Fluor^®^ 488) (Abcam, ab150077), Alexa Fluor 647 Mouse Anti-Human STING antibody (BD bioscience, 564836), MAVS (D5A9E) Rabbit mAb (PE Conjugate) (Cell Signaling Technology, 18930), PE Mouse Anti-Human TRIF (BD bioscience, 566354), Human/Mouse RelA/NF-kB p65 APC-conjugated Antibody (R&D, IC5078A), PE Mouse anti-NF-κB p65(BD bioscience, 558423), Alexa Fluor^®^ 647 Mouse Anti-Human IRF-3 (BD bioscience, 566347), Phospho-IRF-3 (Ser396) (D6O1M) Rabbit mAb (PE Conjugate) (Cell Signaling Technology, 83611). Samples were washed and analyzed by FACS (Beckman Cytoflex).

For dendritic cell staining, the cell suspensions were incubated for 15min at 4°C with Human Hematopoietic Lineage Antibody Cocktail, FITC (eBioscience, 22-7778-72), HLA-DR-PerCP-Cy5.5 (BD bioscience, 339205), CD11c-PE (555392) and CD123-APC (eBioscience, 17-1239-42). For T cell staining, the cell suspensions were incubated for 15 min at 4°C with CD3-FITC (BD bioscience, 557832), CD4-PE (BD bioscience, 555346) and CD8-APC (BD bioscience, 566852). For B cell staining, the cell suspensions were incubated for 15 min at 4°C with CD3-APC-CyTM7 (BD bioscience, 557832), CD19-PerCP-Cy5.5 (BD bioscience, 561295), CD38-APC (BD bioscience, 555462), CD45-FITC (BD bioscience, 555482), and CD138-PE (BD bioscience, 550805).

### 2.5 Statistical analysis

The database was built by using Epidata 3.1 software and data were double entered by two trained data entry staff. Statistical analysis were implemented with SAS 9.4 and SPSS 18.0 software. Continuous variables were expressed as mean ± standard deviation (SD), proportions or geometric mean concentrations (GMCs) with 95% CI as appropriate. Categorical variables were expressed in frequency (%). Statistical methods included Chi-square test, Fisher's exact test, One-Way ANOVA test, with *P* < 0.05 considered statistically significant. Linear regression model, analysis of variance, mediation effect and moderation effect analysis were used to evaluate the effect of folic acid supplementation on non/hypo-response to HepB and innate immune mediators in infants and the relationship between various factors.

## 3 Results

### 3.1 Baseline data of pregnant HBsAg-positive mothers and neonates

A total of 332 mother-neonate pairs and 253 mother-infant pairs were recruited in this cohort study. One HBsAg-positive infant was excluded. All infants received standardized HepB immunoprophylaxis, including three-dose recombinant HepB (administered within 12 h after birth and at 1 and 6 months) combined with hepatitis B immunoglobulin (HBIG) within 12 h after birth. The rate of loss to follow-up was 23.80% (79/332). The key demographic, clinical characteristics did not differ between the infants retained in the study and the loss to follow-ups. According to the time of starting folic acid supplementation, the pregnant woman was divided into non-supplementation group, pre-pregnancy supplementation group and post-pregnancy supplementation group (Supplementary Table 1). Folic acid supplementation included folic acid supplementation alone, iron folic acid supplementation and multivitamin supplementation with folic acid. The follow-up rate did not differ among three groups. Critical demographic parameters remained balanced among the three groups, thereby strengthening the comparability framework.

The demographics and obstetrics characteristics were compared in Table 1. None of the 253 neonates born with any malformations. Notably, the post-pregnancy supplementation group demonstrated superior socioeconomic status, with significantly higher monthly household income compared to both non-supplementation and pre-pregnancy supplementation group (*P* = 0.025). The pre-pregnancy supplementation group showed a prolonged gestational duration relative to other groups (*P* = 0.009).

**Table 1:** The demographics and obstetrics characteristics of HBsAg-positive mothers and their offspring.

**Characteristic**	**Non-supplementation group (*n* = 20)**	**Pre-pregnancy supplementation group (*n* = 70)**	**Post-pregnancy supplementation group (*n* = 163)**	***P***
**HBsAg positive mothers**				
**Age (year)**	28.83 ± 3.80	30.77 ± 3.66	30.46 ± 4.37	0.353
**Education status** ***n*****(%)**				
High school and below	9 (45.00%)	25 (35.71%)	70 (42.94%)	0.626
Technical school/secondary school	5 (25.00%)	29 (41.43%)	54 (33.13%)	
College and above	6 (30.00%)	16 (22.86%)	39 (23.93%)	
**Monthly household income** ***n*****(%)**				
Less than 5,000 RMB	2 (10.00%)	13 (18.57%)	41 (25.15%)	0.025^*^
5000–10,000 RMB	2 (10.00%)	35 (50.00%)	77 (47.24%)	
More than 10,000 RMB	1 (5.00%)	17 (24.29%)	19 (11.66%)	
Refuse to answer	15 (75.00%)	5 (7.14%)	26 (15.95%)	
**Type of medical insurance** ***n*****(%)**				
New rural cooperative	0 (0.00%)	6 (9.23%)	20 (12.27%)	0.923
Medical insurance for urban workers or residents	8 (61.54%)	36 (55.38%)	87 (53.37%)	
Self-pay	4 (30.77%)	15 (23.08%)	36 (22.09%)	
Other types	1 (5.39%)	8 (12.31%)	20 (12.27%)	
**HBV DNA (IU/ml)** ***n*****(%)**				
< 2 × 10^5^	16 (80.00%)	61 (87.14%)	139 (85.28%)	0.726
≥2 × 10^5^	4 (20.00%)	9 (12.86%)	24 (14.72%)	
**HBeAg** ***n*****(%)**				
–	7 (35.00%)	28 (40.00%)	68 (41.72%)	0.838
+	13 (65.00%)	42 (60.00%)	95 (58.28%)	
**Antiviral drug use** ***n*****(%)**				
Yes	15 (75.00%)	48 (68.57%)	103 (63.19%)	0.478
No	5 (25.00%)	22 (31.43%)	60 (36.81%)	
**Gestational weeks**				
Mean ± SD	39.05 ± 0.94	39.24 ± 1.04	38.80 ± 1.03	0.009^*^
**Delivery mode** ***n*****(%)**				
Vaginal delivery	32 (69.57%)	41 (61.19%)	80 (57.14%)	0.324
Cesarean delivery	14 (30.43%)	26 (38.81%)	60 (42.86%)	
**Neonates**				
**Sex n(%**)				
Female	10 (50.00)	41 (58.57)	89 (54.60)	0.755
Male	10 (50.00)	29 (41.43)	74 (45.40)	
**Apgar score**	10.00 ± 0.00	9.99 ± 0.08	9.99 ± 0.03	0.515
**Body weight (g)**	3339.75 ± 483.20	3407.21 ± 442.26	3284.20 ± 386.50	0.111
**Body length (cm)**	50.05 ± 3.38	49.73 ± 2.25	49.73 ± 1.59	0.785

One-way analysis of variance and Chi-square and Fisher's exact test were used to compare the differences among three groups. ^*^In the table indicate statistical significance (*p* < 0.05).

### 3.2 The association between maternal folic acid supplementation and innate immunity of neonates

Folic acid supplementation timing did not influence neonatal immune cell proportions. As shown in Table 2, the proportion of immune cells remained stable in neonates among the pre-pregnancy supplementation group, the post-pregnancy supplementation group, and the non-supplementation group.

**Table 2:** The effects of different folic acid supplementation on immune cells proportion of cord blood (%).

**Immune cells proportion**	**Non-supplementation group (*n* = 20)**	**Pre-pregnancy supplementation group (*n* = 70)**	**Post-pregnancy supplementation group (*n* = 163)**	***P***
DCs	1.13 ± 0.88	1.75 ± 2.43	2.40 ± 4.34	0.705
mDCs	0.75 ± 0.86	0.64 ± 0.93	1.52 ± 3.53	0.856
pDCs	0.47 ± 0.35	0.62 ± 1.05	0.51 ± 0.55	0.398
T cells	31.93 ± 19.25	22.13 ± 15.56	28.20 ± 18.03	0.627
CD8^+^ T cells	5.46 ± 4.50	4.94 ± 3.96	6.05 ± 4.41	0.297
CD4^+^ T cells	15.21 ± 11.20	12.29 ± 9.47	16.95 ± 11.05	0.092
B cells	8.55 ± 5.89	5.37 ± 3.59	7.95 ± 4.85	0.713
Plasma cells	0.83 ± 2.74	0.18 ± 0.25	0.59 ± 2.35	0.490

One-way analysis of variance were used to compare the differences of immune cells proportion among three groups.

Folic acid supplementation timing significantly affected STING and pNF-κB expression levels in cord blood. Table 3 shows the expression level of STING (*P* = 0.005) and pNF-κB (*P* = 0.010) in cord blood were significantly different among three folic acid supplementation groups. *Post hoc* analyses confirmed that the pre-pregnancy supplementation group had significantly higher levels of STING and pNF-κB level compared to non-supplementation group and post-pregnancy supplementation group.

**Table 3:** The effects of different folic acid supplementation on the expression levels of innate immune mediators in cord blood (%).

**Innate immune mediators**	**Non-supplementation group (*n* = 20)**	**Pre-pregnancy supplementation group (*n* = 70)**	**Post-pregnancy supplementation group (*n* = 163)**	***P***
MAVS	78.73 ± 24.20	85.23 ± 18.91	85.31 ± 20.34	0.387
TRIF	90.81 ± 13.38	95.96 ± 7.03	95.63 ± 10.66	0.113
MyD88	75.93 ± 23.33	83.20 ± 22.32	81.42 ± 22.65	0.448
STING	36.53 ± 37.95	66.70 ± 34.31	62.45 ± 36.86	0.005^*^
NF-κB	78.70 ± 16.15	78.59 ± 24.10	78.51 ± 26.65	0.999
pNF-κB	31.00 ± 22.70	50.87 ± 28.08	49.45 ± 26.46	0.010^*^
IRF3	98.83 ± 3.03	97.24 ± 12.48	97.21 ± 13.88	0.868
pIRF3	21.99 ± 23.58	30.36 ± 27.60	23.14 ± 23.18	0.121

One-way analysis of variance were used to compare the differences of innate immune molecular expression levels among three groups. ^*^In the table indicate statistical significance (*p* < 0.05).

### 3.3 The effects of STING and pNF-κB expression level in cord blood on the anti-HBs level of infants

Neonatal innate immune mediators STING and pNF-κB expression levels varied significantly among folic acid supplementation groups. As shown in Table 3, significantly differences in STING (*P* = 0.005) and pNF-κB (*P* = 0.010) expression levels were found across the three folic acid supplementation groups. This finding promoted further investigation into whether these differences play a role in infant antibody production.

High pNF-κB expression in cord blood was associated with improved seropositive rates for anti-HBs in infants. In this study, all infants received three doses of HepB vaccination. Anti-HBs levels were measured 1–3 months after full vaccination of HepB by CMIA. The effect of different STING and pNF-κB expression level in neonates cord blood on the anti-HBs level of 7–9 months old infants were compared in Table 4. Infants were stratified into high and low expression groups based on the median expression level of STING and pNF-κB in cord blood. The seropositive rate (anti-HBs >10 mIU/ml) in the high pNF-κB expression group was significantly higher than in the low pNF-κB expression group (*P* = 0.036). However, no significant differences in anti-HBs levels was observed between high and low STING expression groups (Table 4). Strong seropositive rates (anti-HBs >100 mIU/ml) were also analyzed, but no significant differences were found between groups.

**Table 4:** The effects of STING and pNF-κB expression in cord blood on anti-HBs level of infants born to HBsAg-positive mothers.

**Different level of innate immune mediators**	**Anti-HBs level in 7–9 months old infants (%)**					**Seropositive rate (%)**	***P***	**Strong seropositive rate (%)**	***P***	**GMC (mIU/ml) (95% *CI*)**	***P***
	**Total**	<**10**	**10–**	**100–**	**1,000–**						
**STING**											
High	127 (50.20)	5 (45.45)	7 (46.67)	49 (52.69)	66 (49.25)	122 (96.06)	0.748	115 (90.55)	0.663	2,892.92 (2,153.98–3,632.09)	0.836
Low	126 (49.80)	6 (54.55)	8 (53.33)	44 (47.31)	68 (50.75)	120 (95.24)		112 (88.89)		2,780.77 (2,006.42–3,555.12)	
**pNF-**κ**B**											
High	127 (50.20)	3 (27.27)	5 (33.33)	44 (47.31)	75 (55.97)	124 (97.64)	0.120	119 (93.70)	0.036^*^	3,153.40 (2,394.98–3,911.82)	0.240
Low	126 (49.80)	8 (72.73)	10 (66.67)	49 (52.69)	59 (44.03)	118 (93.65)		108 (85.71)		2,518.28 (1,767.26–3,269.20)	

1. Chi-square test and Fisher's exact test were used to compare the differences of seropositive rate and strong seropositive rate between high and low expression groups.

2. One-way analysis of variance was used to compare the GMCs of anti-HBs between high and low expression groups.

3. ^*^In the table indicate statistical significance (*p* < 0.05).

### 3.4 The impact of maternal preconception folic acid supplementation on anti-HBs in infants born to HBsAg-positive mothers

Preconception folic acid supplementation was associated with significantly higher anti-HBs levels in infants. The GMCs of anti-HBs differed significantly among the non-supplementation group, the preconception supplementation group and the post-pregnancy supplementation group (*P* = 0.006). Specifically, infants in the preconception supplementation group had significantly higher anti-HBs levels compared to the other two groups, while no significant diferences were observed between the non-supplementation group and post-pregnancy supplementation group (Table 5).

**Table 5:** The effects of folic acid supplementation on anti-HBs level of infants born to HBsAg-positive mothers.

**Folic acid supplement-ation subgroup**	**Anti-HBs level in 7–9 months old infants (%)**					**Seropositive rate (%)**	***P***	**Strong seropositive rate (%)**	***P***	**GMC (mIU/ml) (95% *CI*)**	***P***
	**Total**	<**10**	**10–**	**100–**	**1,000–**						
Non-supplementation group	20	0 (0.00)	1 (5.00)	11 (55.00)	8 (40.00)	20 (100.00)	0.529	19 (95.00)	0.472	1,554.30 (888.42–2,220.13)	0.006^*^
Pre-pregnancy supplementation group	70	2 (2.86)	3 (4.29)	19 (27.14)	46 (65.71)	68 (97.14)		65 (92.86)		4,178.30 (2,851.52–5,505.23)	
Post-pregnancy supplementation group	163	9 (5.52)	11 (6.75)	63 (38.65)	80 (49.08)	154 (94.48)		143 (87.73)		2,418.43 (1,838.00–2,998.91)	

1. Chi-square test and Fisher's exact test were used to compare the differences of seropositive rate and strong seropositive rate between high and low expression groups.

2. One-way analysis of variance was used to compare the GMCs of anti-HBs between high and low expression groups.

3. ^*^In the table indicate statistical significance (*p* < 0.05).

Preconception folic acid supplementation independently predicted higher anti-HBs levels in infants. A linear regression model was used to assess the effects of preconception folic acid supplementation on infants anti-HBs levels. The definition of the variables was displayed in Supplementary Table 2. After adjustment of potential confounders, preconception folic acid supplementation was significantly associated with higher anti-HBs levels in infants, as evidenced by an adjusted β-value of 2063.55 (*P* = 0.001, 95%*CI*: 774.358–3,174.135). No significant associations were found in other variables (*P* > 0.05) (Table 6).

**Table 6:** The impact of preconception folic acid supplementation on anti-HBs level of infants born to HBsAg-positive mothers.

**variable**	**β-value**	**SE**	**95%** ***CI***		**Adjusted β-value**	***t***	***P***
			**Lower**	**Upper**			
Preconception folic acid supplementation	2,063.550	609.112	774.358	3,174.135	0.203	3.241	0.001^*^
Maternal HBV DNA	0.000	0.000	0.000	0.000	−0.100	−0.817	0.415
Maternal HBeAg	0.054	0.041	−0.027	0.134	0.161	1.318	0.189
Maternal antiviral drug use	470.928	572.735	−657.301	1,599.158	0.050	0.822	0.412
Maternal education status	332.547	345.865	−348.772	1,013.866	0.061	0.961	0.337
Monthly household income	−154.194	282.357	−710.408	402.020	−0.036	−0.546	0.586
Delivery mode	−856.150	555.128	−1949.696	237.397	−0.098	−1.542	0.124
Neonatal prematurity	−1,647.618	2,152.162	−5,887.158	2,591.922	−0.048	−0.766	0.445
Neonatal birth weight	−494.821	1,003.049	−2,470.724	1,481.082	−0.031	−0.493	0.622
STING expression in cord blood	808.701	755.632	−679.817	2,297.218	0.069	1.070	0.286
pNF-κB expression in cord blood	938.892	1,015.221	−1,060.989	2,938.773	0.059	0.925	0.356

^*^In the table indicate statistical significance (*p* < 0.05).

### 3.5 Effects of folic acid supplementation before pregnancy on anti-HBs level of infants by pNF-κB expression in cord blood

Cord blood pNF-κB expression did not mediate the association between maternal preconception folic acid supplementation and infant anti-HBs levels. A mediation model was performed to explore the potential role of cord blood pNF-κB expression in the observed association between maternal preconception folic acid supplementation and higher anti-HBs levels in infants. After adjusting for the same adjusted factors as above linear regression model, no significant association was found between neonates pNF-κB expression and infant anti-HBs levels (*P* > 0.05) (Supplementary Table 3, Supplementary Figure 1). This indicated that neonatal pNF-κB expression did not act as mediators in this relationship.

No moderation effects were observed between maternal folic acid supplementation, neonatal pNF-κB expression, and infant anti-HBs levels. A moderation effects model was also performed to assess potential interactions. There were no moderation effect between “maternal folic acid supplementation” and “neonatal pNF-κB expression” or “infants anti-HBs level” and “neonatal pNF-κB expression” (*P* > 0.05) (Supplementary Table 4).

## 4 Discussion

As an important water-soluble vitamin, folic acid is closely related to cell proliferation and differentiation and plays important role in fetus development. WHO recommendations indicate that women should take a folic acid supplement during 8 weeks before and 8 weeks after conception (18). Therefore, it is still unknown whether and how maternal folic acid supplementation before pregnancy and infants innate immunity are related, especially in infants born to HBsAg-positive mothers. In our study, the innate immune mediators and anti-HBs level of offspring in pre-pregnancy group were observed, with significant differences compared to non-supplementation group and post-pregnancy supplementation group. As expected, the expression level of STING and pNF-κB and anti-HBs level were increased in pre-pregnancy group and high pNF-κB expression in cord blood can increase seropositive rates of HepB in infants.

The Chinese government has launched the National Free Preconception Health Examination Project (NFPHEP) since 2010, in which, women planning a pregnancy within 6 months are freely provided daily 0.4 mg folic acid and recommended to take folic acid starting from 3 months before conception until the end of the first trimester in order to prevent NTDs (19). Moreover, the US Preventive Service Task Force recommended women should take a daily dosage of 0.4–0.8mg folic acid when planning to get pregnancy (20). Most guidelines recommend folic acid supplementation starting at least 3 months before conception, but the end time is inconsistent (19, 21). In Japan, 45.1% of women initiated folic acid supplementation before pregnancy (22). It has been reported that the percentage of folic acid supplementation in pregnant women of general population were 74.7–94.5% (23–25) in the mainland of China, among them 52.3%−54.37% take folic acid before conception (23, 26). For HBsAg-positive mothers, previous study showed the percentage of folic acid supplementation during the first trimester was 63.59% (27). However, when considered the pre-conceptional periods, an equally important period for fetus development, the percentage of folic acid supplementation was 15.38% (27). In our study, only 27.67% pregnant women took folic acid before conception. Previous studies have shown that the percentage of folic acid supplementation in pregnant women was associated with educational level, the women with lower educational level are less likely to take folic acid supplementation than those with higher educational level (28). In this study, approximately half of HBsAg-positive mother has a high school education or below, which lead to low percentage of folic acid supplementation before and during pregnancy. In this study, we observed that the folic acid supplementation groups (including both pre-pregnancy and post-pregnancy supplementation groups) had a higher average monthly household income compared to the non-supplementation group. This finding is consistent with previous studies, which have reported that mothers with lower educational attainment and lower household income are less likely to take folic acid supplements before pregnancy. This disparity may be attributed to insufficient access to preventive healthcare during early pregnancy and a lack of awareness regarding the importance of folic acid supplementation among these populations (29).

The immune system in the newborn is immature and may be affected by several external factors. However, the evidence of maternal folic acid supplementation of HBsAg-positive mothers in the innate immunity of offspring is scarce. In our study, we observed innate immunity mediators and HepB vaccine responses in offspring across different folic acid supplementation groups. Notably, the pre-pregnancy supplementation group exhibited significantly higher expression of STING and pNF-κB in cord blood compared to the non-supplementation and post-pregnancy supplementation groups. It is reported that maternal folic acid supplement during pregnancy was associated with changes in DNA methylation located in genes implicated in embryonic development, immune response and cellular proliferation in the offspring (30). STING is an endoplasmic reticulum dimeric adaptor protein and expressed in various immune cells and acts as a major regulator of type I interferon release and innate immune response (31). Moreover, a major transporter of folate nutrients can recognize and transport cyclic dinucleotides (CDNs), thereby can act as second messengers to activate STING and elicit broad downstream responses (32). This suggests a potential mechanistic link between folate metabolism and STING pathway activation. Folic acid has been shown to ameliorate the inflammatory response in LPS-activated THP-1 macrophages, and this effect is dependent on the NF-κB signaling pathway (33). In this study, maternal folic acid supplementation affect the expression of STING pathway and the downstream molecule NF-κB, which was highly expressed in T cells. T cells constitute approximately half of cord blood mononuclear cells, and while no significant differences in T cell proportions were observed among the three groups, the functional impact of maternal folic acid supplementation on T cells cannot be ruled out. Specifically, folic acid-mediated changes in DNA methylation and STING pathway activation may enhance T cell responsiveness or alter their functional profile without affecting their overall proportion. These findings suggest that maternal folic acid supplementation may influence innate immunity in offspring by modulating the STING/pNF-κB signaling axis, potentially through epigenetic regulation and folate-dependent metabolic pathways. Further research is needed to elucidate the precise mechanisms by which folic acid impacts immune cell function and vaccine response in neonates.

HBV infection remains a global public health burden. The Asia-Pacific region having half of the 20 most heavily burdened countries and experiences a greater challenge of HBV infection (34). HepB vaccination is the most effective way to prevent HBV infection. The immunogenicity of HepB depends on synergistic interactions between innate and adaptive immune systems, ultimately manifested through anti-HBs production. Previous study focus on the relationship between maternal folic acid supplementation during pregnancy and infant vaccine responsiveness at 11–12 months (27) and 5 years (35), critical knowledge gaps persist regarding early immune maturation. Notably, the 1–2 month post-vaccination window, a pivotal phase for assessing primary humoral response, has been underexplored in this high-risk populations. Our study analyzed 253 HBsAg-positive mother-infant pairs, among them, 25 infants were non/hypo-responders (anti-HBs titres < 100 mIU/ml) and the rate of non/hypo-response was 9.88%. Comparatively, it is reported that the non/hypo-response rates in this population was 12.2%−24.4% at 7 months and 28.4%−30.2% at 12 months (36, 37). It can be seen that the non/hypo-response rate of infants born to HBsAg-positive mothers has a downward trend, as China has a large population base, which remains an important public health issue. For poor responders of HepB, additional interventions are urgently needed to protect them from HBV.

The association between the expression of STING and pNF-κB in neonates and anti-HBs level in infants was also explored in this study. The seropositive rate of infants in the high pNF-κB expression group was significantly higher than low expression group and there are high anti-HBs titer trends in high pNF-κB expression group. It is reported that the STING/NF-κB pathway was one of the important pathways to activate innate immunity (38), which means that pNF-κB may promote HepB vaccine response in infants.

Whether folic acid supplementation affects infants antibody level were further explored. Compared with non-supplementation group, the anti-HBs level of infants in the supplementation group including pre-pregnancy group and post-pregnancy group were significantly higher. The linear regression model also showed that take folic acid before conception was associated with high anti-HBs levels. At present, many researchers are studying on improving the nutritional status before conception and during pregnancy in order to promote the development of immune response to vaccine. Previous study had shown that giving nutritional supplements to nutritionally vulnerable pregnant mothers in African improves antibody response to vaccination in early infancy (39). In addition, maternal folic acid supplementation during pregnancy could improve the persistence of protective antibodies in infants, this improvement may due to folic acid deficiency can reduce iron absorption and lead to anemia, and iron deficiency anemia may influence non-specific immunity. Moreover, folic acid may also affect the persistence of anti-HBs through methyl mechanisms (35).

Furthermore, mediation model and moderate model were constructed to explore the relationship among maternal folic acid supplementation, neonate pNF-κB expression and infants anti-HBs level. However, there were no mediation effect and no moderation effect in these models. The “black box” between the maternal folic acid supplementation and infants anti-HBs were not clarified yet in this study. There are complex mechanisms in epidemiological studies. pNF-κB may not be the only mediator in this study, and there were no statistical difference may due to the sample size. We hypothesized that the effect of folic acid supplementation on immune-related genes in infants may be one of the underlying mechanism for the difference in protective antibody levels three folic acid groups. Further studies were needed to increase the sample size or add indicators to explore and evaluate biological or social mechanisms between maternal folic acid supplementation and infants anti-HBs level.

The study assessed that HBsAg-positive mothers who in-took folic acid before conception, the expression of innate immune mediators tend to increase in cord blood and their infants tend to have higher protective antibody titers after HepB vaccination. Moreover, higher expression of pNF-κB in cord blood might substantially increase the seropositive rate of infants. Therefore, our findings suggested that folic acid supplementation should be taken before conception period for HBsAg-positive mothers who are planning to become pregnant.

Our findings may have some clinical implications for the relevant policies and practices of folic acid supplementation in HBsAg-positive mothers in China to some extent. Our results highlighted that the critical window for increasing offspring anti-HBs level is the prenatal period. These findings might be useful for maternal health care of HBsAg-positive mothers considering implementation of folic acid supplementation to reduce the risk of infants non/hypo-response to HepB and reach the goal of eliminating viral hepatitis as a major public health threat by 2030 set by WHO. Further studies are required to elucidate whether folic acid supplements be used consistently over the periods demarcated and whether HBsAg-positive mothers should be asked to take the supplements from preparation to the end of pregnancy or only up to a certain point.

In our ambispective cohort study, the information if innate immunity of neonates were collected at the time of delivery, while the information of maternal folic acid supplementation were collected retrospectively and the anti-HBs level of infants were collected prospectively. At the same time, this study also assessed whether there is a causal relationship among maternal folic acid supplementation, innate immune mediators protein expression level and anti-HBs levels, which has not been studied yet.

Regarding the limitations, we collected folic acid supplement information by questionnaire survey and did not measure the maternal folic acid levels. The use of questionnaires might introduce potential recall bias, but it remains one of the most effective investigation methods in epidemiological studies and can achieve the purpose of this study to a certain extent. To minimize recall bias, questionnaire data were cross-referenced with maternal pregnancy records and prenatal examination reports during data collection. Follow-up studies were needed to collect the dosage of the folic acid supplementation and collect peripheral blood in early, middle and late pregnancy to measure the serum folic acid levels to investigate the dose–response associations before and during prenatal periods. Besides, the relationship among maternal folic acid supplementation, neonate pNF-κB expression and infants anti-HBs level were not elucidated in this study. In China, the National Pre-pregnancy Health Check Project provides free folic acid supplementation to women planning to conceive within 6 months, recommending initiation 3 months before conception and continuation through early pregnancy. As a result, the number of participants in the non-supplementation group was relatively small, leading to an imbalance in group sizes. Additionally, the overall sample size of this study was limited, resulting in suboptimal statistical power. Nevertheless, our findings provide preliminary insights and offer valuable clues for informing folic acid supplementation policies targeting HBsAg-positive mothers. A larger sample size studies were needed to confirm the stability of our results in the future.

## 5 Conclusion

In summary, preconception folic acid supplementation may facilitate the expression of STING and pNF-κB in neonates and anti-HBs level in infants. Meanwhile, high pNF-κB expression at birth can lead to higher seropositive rate of HepB in infants aged 7–9 months.

## Data Availability

The original contributions presented in the study are included in the article/Supplementary material, further inquiries can be directed to the corresponding authors.
