# Supplementary material for: Effects of folic acid supplementation before conception on innate immunity and anti-HBs levels of offspring born to HBsAg-positive mothers
Source: Front Nutr. 2025 Apr 23;12:1526053. doi: 10.3389/fnut.2025.1526053 (PMC12055552; doi:10.3389/fnut.2025.1526053)
Supplement: Supplementary file 1 [file Table_1.docx]

Supplementary materials

### Table 1. Time distribution of folic acid supplementation in HBsAg positive mothers

|  | N | End folic acid supplement time | | | |
| --- | --- | --- | --- | --- | --- |
|  |  | before pregnancy | during 1st trimester | during 2nd trimester | during 3rd trimester |
| Non-  supplementation group | 20 | / | / | / | / |
| Preconception supplementation group | 70 | 7(10.00) | 11(15.71) | 8(11.43) | 44(62.86) |
| Post-pregnancy supplementation group | 163 | / | 60(36.81) | 28(17.18) | 75(46.01) |

Note: Chi-square and Fisher's exact test were used to compare the distribution between two groups.

Table 2. The definition of variable in linear regression model.

| Variables | Definition |
| --- | --- |
| Infant anti-HBs level (Y) | continuous variable (mIU/mL) |
| Preconception folic acid supplementation (X1) | 0=No, 1=Yes |
| Maternal HBV DNA(X2) | 0=“＜2×10^5^ IU/ml”, 1=“≥2×10^5^ IU/ml” |
| Maternal HBeAg(X3) | 0=HBeAg negative, 1=HBeAg positive |
| Maternal antiviral drug use(X4) | 0=No, 1=Yes |
| Maternal education status(X5) | 0=High school degree and below, 1=Technical school/ secondary school,  2= college and above |
| Monthly household income(X6) | 0= Less than 5000 RMB, 1= 5 000 to 10 000 RMB, 2= More than 10 000 RMB, 3= Refuse to answer |
| Delivery mode (X7) | 0= Cesarean delivery 1=Vaginal delivery |
| Neonatal prematurity(X8) | 0=Term infant, 1=Premature infant |
| Neonatal body weight(X9) | 0=“2500-4000g”, 1=“＞4000g” |
| STING expression in CBMCs(X10) | continuous variable |
| pNF-κB expression in CBMCs(X11) | continuous variable |


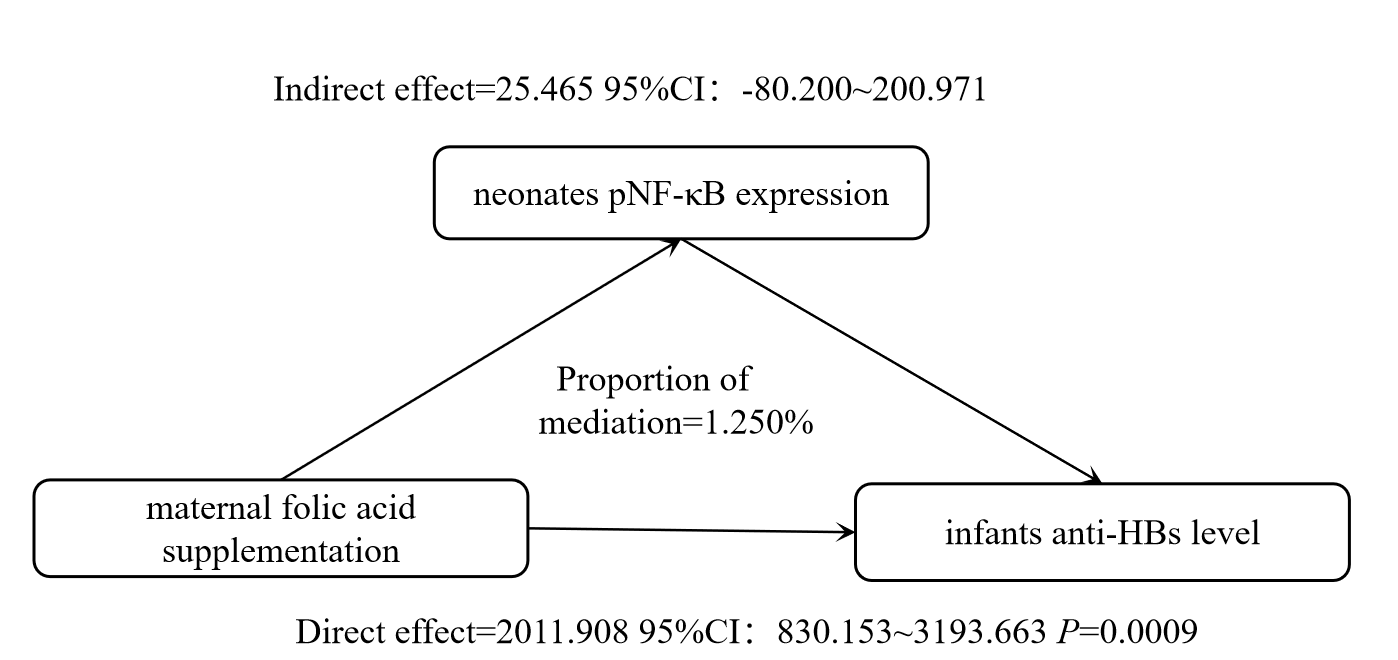


Figure 1. The mediation effect of neonates pNF-κB expression between maternal preconception folic acid supplementation and anti-HBs level among infants.

Table 3. The mediation effect of neonates pNF-κB expression between maternal preconception folic acid supplementation and anti-HBs level among infants.

|  | Effect | se | 95% CI | | Proportion of mediation |
| --- | --- | --- | --- | --- | --- |
|  |  |  | lower | upper |  |
| Total effect | 2037.373 | 599.223 | 857.227 | 3217.520 |  |
| Direct effect | 2011.908 | 600.029 | 830.153 | 3193.663 |  |
| Indirect effect | 25.465 | 67.918 | -80.200 | 200.971 | 1.250% |

Table 4. The moderation effects of neonates pNF-κB expression between maternal preconception folic acid supplementation and anti-HBs level among infants.

|  | coeff | se | *t* | *p* | 95% CI | |
| --- | --- | --- | --- | --- | --- | --- |
|  |  |  |  |  | lower | upper |
| constant | 2837.073 | 265.274 | 10.695 | 0 | 2314.607 | 3359.54 |
| Preconception folic acid supplementation | 2011.968 | 601.858 | 3.343 | 0.001 | 826.586 | 3197.349 |
| neonates pNF-κB expression | 907.401 | 984.185 | 0.922 | 0.357 | -1030.989 | 2845.791 |
| Int | -4.735 | 2175.756 | -0.002 | 0.998 | -4289.968 | 4280.498 |
